# Unfolding Behavior and Conformational Changes Under Different Denaturing Conditions of MAPK 1 (MEK1)

**DOI:** 10.3390/biom16060845

**Published:** 2026-06-09

**Authors:** Maria Gabriela Álvarez-Rodríguez, Sonia Vega, Felipe Hornos, Adrian Velazquez-Campoy, Bruno Rizzuti, José L. Neira

**Affiliations:** 1 Instituto de Biotecnologia Sanitaria de Elche (IDIBE), Universidad Miguel Hernández, 03202 Elche, Spain; m.alvarezr@umh.es (M.G.Á.-R.); fhornos@umh.es (F.H.); 2 Instituto de Biocomputación y Física de Sistemas Complejos (BIFI), Universidad de Zaragoza, 50018 Zaragoza, Spain; svega@bifi.es (S.V.); adrianvc@unizar.es (A.V.-C.); bruno.rizzuti@cnr.it (B.R.); 3 Instituto de Investigación Sanitaria Aragón (IIS Aragón), 50009 Zaragoza, Spain; 4 Centro de Investigación Biomédica en Red de Enfermedades Hepáticas y Digestivas (CIBEREHD), 28029 Madrid, Spain; 5 Departamento de Bioquímica y Biología Molecular y Celular, Universidad de Zaragoza, 50009 Zaragoza, Spain; 6 CNR-NANOTEC, SS Rende (CS), Department of Physics, University of Calabria, 87036 Rende, Italy

**Keywords:** MEK1, conformational stability, circular dichroism, fluorescence, differential scanning calorimetry, constraint network analysis

## Abstract

Protein kinases have key roles in cells as they regulate diverse signal transduction pathways. Mitogen-activated protein kinase (MAPK) signaling route modulates several processes, such as cell proliferation, cell programming, metabolic changes and stress responses. Within the group of proteins participating in this pathway, the MAPK kinase (MEK1) is a dimeric, 393-residue-long, dual-specificity protein kinase that phosphorylates both tyrosine and threonine residues. In this study, we explored the conformational changes occurring during the unfolding of MEK1, by using orthogonal biophysical techniques. Intrinsic fluorescence, extrinsic 8-anilinonapthalene-1-sulfonic acid (ANS) fluorescence, dynamic light scattering (DLS), and far-ultraviolet (UV) circular dichroism (CD) showed that the protein acquired a native-like conformation within a narrow pH range (8.0 to 9.0). Urea and guanidinium hydrochloride (GdmCl) denaturations followed by intrinsic and ANS fluorescence and far-UV CD, at pH 8.1, where the protein acquired a native-like conformation, showed that: (i) the apparent conformational stability of isolated MEK1 was low; and (ii) the unfolding occurred through the presence of intermediates. The presence of several unfolding intermediates was also evidenced through: (i) differential scanning calorimetry (DSC) in the absence of the ligand ATP; and (ii) unfolding simulations with the help of computational techniques based on constraint network analysis (CNA). We propose that the apparent low stability of this protein was related to its flexibility and modulates its ability to interact with diverse molecular partners.

## 1. Introduction

Phosphorylation is a reversible post-translational modification (PTM) that acts as a modulator of several protein-dependent functional routes, including, among others, metabolism, cell division, DNA replication and apoptosis. The so-called protein kinases carry out this PTM, and they are involved in diverse signal transduction pathways within the cells [1,2]. Around forty per cent of proteins are substrates of kinases, including many kinases themselves [3]. Due to their significant role in signaling, misregulation of protein kinases is a feature of many diseases.

Among the several protein pathways capable of regulating cell growth, the mitogen-activated protein kinase (MAPK) cascade is critical in modulating cellular differentiation, proliferation, death and apoptosis, as well as metabolic rewiring, inflammatory controls and the cellular response to stress [4,5,6,7]. Under physiological conditions, the MAPK signaling cascade is tightly modulated by cytokines, receptor tyrosine kinases and growth factors. The MAPK cascade has three major pathways: the extracellular signal-regulated kinase (ERK) or MAPK; the c-Jun N-terminal kinase/stress-activated protein kinase (JNK/SAPK); and the p38 pathway [7]. Each route has distinct functions: the ERK-MAPK signaling cascade is involved in cell proliferation, cell survival and the underlying metabolic variations; the JNK pathway intervenes in cell survival and apoptotic processes; and the p38 signaling cascade intervenes in cell differentiation, survival, proliferation, apoptosis, development, inflammation and stress responses [6,8,9].

The ERK-MAPK pathway is considered the classical MAPK signaling cascade [9,10,11]. It is constituted by the following proteins: a MAPK extracellular signal-regulated kinase (such as ERK1 and ERK2); a MAPK kinase, also known as MAPKK (such as MEK1 and MEK2); and a MAPKK kinase (such as Raf or MKKK/MAPKKK). MEK1 is a dual-specificity kinase that phosphorylates the downstream target ERK/MAPK on specific threonine and tyrosine amino acids by using ATP as the phosphoryl source; it is activated by the concomitant phosphorylation of two serine residues in the so-called activation loop, namely Ser218 and Ser222, by the upstream Raf protein [2,4,12,13].

**Figure 1:**
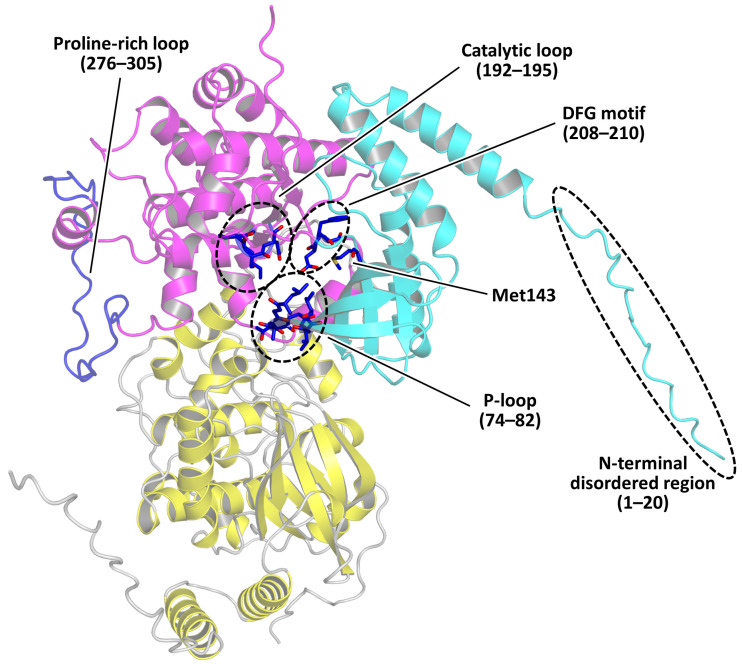
Model of dimeric MEK1 structure. One monomer is shown in a single color (yellow), and the other monomer in different colors. N-terminal lobe (cyan, residues 1–145), and C-terminal lobe (magenta, residues 146–393), including the proline-rich loop (violet, residues 276–305). Some structures delimiting the ATP-binding site are shown in stick representation (blue): the P-loop (residues 74–82), residue Met143 in the hydrophobic pocket, the catalytic loop (residues 192–195), and the DFG motif (residues 208–210). The relatively long N-terminal tail (residues 1–20) is disordered and may assume different conformations. Model obtained by using AlphaFold-Multimer, on the basis of a variety of MEK1 structures deposited in the Protein Data Bank.

From a structural point of view, as shown in Figure 1, MEK1 is a dimeric protein. Each monomer contains 393 residues and is formed by two lobes: the N-terminal and C-terminal lobes [14]. The ATP binding site is sandwiched between both regions, as shown in PDB entry 3W8Q [15], which contains MEK1 in complex with an ATP-γS (i.e., a non-hydrolyzable ATP analog, with one of the oxygens of phosphate changed to sulfur) molecule bound. The N-terminal lobe contains a short α-helix (residues 118–122) and the P-loop (residues 74–82). The C-terminal lobe contains the activation segment (residues 210–233) and another α-helix (residues 245–256). The catalytic loop (192–195), hydrophobic pocket (including residue 143), and the DFG motif (residues 208–210) form the critical binding pocket for ATP. The proline-rich loop (residues 276–305) is an important regulatory subdomain, containing Ser298 and Tyr300 phosphorylation sites, required for efficient activation of ERK1 [16]. The dimerization interface spans from the N-terminal lobe to the C-terminal one, including the nucleotide-binding loop, the in-between loop of the third β-strand and an α-helix, the nucleotide-binding loop and another α-helix [17]. MEK1 is one of the main targets for the design of specific drugs to fight cancer. Despite the wealth of knowledge on the design of new compounds against MEK1 [18,19], and in sharp contrast with the accurate structural data available for the well-folded state protein, there is currently no description of its unfolding pathway and the conformational changes occurring during its unfolding reaction. In particular, there is no clue about the presence of folding intermediates whose existence could be interesting to know with the goal of: (i) polishing the design of drugs capable of binding to the MEK1; or (ii) binding to and stabilizing folding intermediates, to block upregulated MEK1. Any information about those intermediates, even if it is not at the detailed structural atomic level, could be useful to achieve those goals.

In this work, we studied the unfolding of MEK1 and its concomitant conformational changes under different pH conditions. When the biophysical properties of the protein, being monitored, reached a plateau, as the pH was changed, we could conclude at which pH values the protein acquired a native-like conformation. And once we had framed that pH range, we could attempt to study the conformational stability of the protein by using different chemical (urea and GdmCl) and thermal denaturations. To that end, we used orthogonal biophysical techniques, namely fluorescence, far-ultraviolet (UV) circular dichroism (CD), dynamic light scattering (DLS) and differential scanning calorimetry (DSC). Furthermore, the unfolding of MEK1 was simulated by using an analysis of the protein constraint network (CNA), which is based on graph theory [20]. All the findings suggested that MEK1 showed low conformational stability and low conformational cooperativity, acquiring a native-like conformation in a narrow pH range (pH 8.0 to 9.0). Unfolding of MEK1, as judged from thermal and chemical denaturations, suggested the presence of intermediates. This presence was also observed by the in silico experiments, which showed that the two protein lobes unfold in a sequential way rather than simultaneously.

## 2. Materials and Methods

### 2.1. Materials

Trizma base, Trizma acid, imidazole, DNase, Roche protease tablets, 8-anilinonapthalene-1-sulfonic acid (ANS), NaCl, Ni^2+^-resin, kanamycin and Amicon centrifugal devices, with a molecular weight cut-off of 10 kDa, were purchased from Sigma (Madrid, Spain). The isopropyl-β-D-1-thiogalactopyranoside was obtained from Apollo Scientific (Stockport, 1 Parkway, Denton, Manchester, UK). The β-mercaptoethanol was from BioRad (Madrid, Spain). The Triton X-100, dialysis tubing with a molecular weight cut-off of 3500 Da, and the SDS protein marker (PAGEmark Tricolor) were from VWR (Barcelona, Spain). Ultrapure urea and guanidinium hydrochloride (GdmCl) were from MP Biomedicals (OH, 9 Goddard, Irvine, CA, USA). Exact concentrations of the urea and GdmCl stock solutions used during chemical denaturations were calculated from their corresponding refractive indexes [21]. The rest of the materials were of analytical grade. Water was deionized and purified on a Millipore system.

### 2.2. Protein Expression and Purification

The vector pET28b (kanamycin resistant), containing the genetic code for the entire MEK1 with a histidine-tag at its N terminus, was a kind gift from Dr. Xuesen Zhang (Department of Histology and Embryology, College of Basic Medical Science, China Medical University, 110122 Shenyang, Liaoning Province, China).

BL21 (DE3) *E. coli* strain (Merck, Madrid, Spain) was transformed with the pET28b vector containing MEK1. Basically, the protein was expressed and purified as described for the deletion variant of MEK1 (residues 62–293) [17,22,23]. Ultraviolet (UV) absorbance was used to determine protein concentrations by employing an extinction coefficient at 280 nm estimated from the number of tyrosines (9) and tryptophans (2) (ε_280nm_ = 22,900 M^−1^ cm^−1^) in the sequence of the MEK1 monomeric species [24,25].

### 2.3. Fluorescence

All fluorescence spectra were collected on a Cary Varian spectrofluorometer (Agilent, Santa Clara, CA, USA), interfaced with a Peltier unit, at 25 °C, and using a 1 cm-pathlength quartz cell (Hellma, Müllheim, Germany). Sample concentration in the pH and chemical denaturation experiments was 4 μM (in protomer units), and the final concentration of the buffer was, in all cases, 50 mM. Under this concentration of the protomer, and taking into account that the dissociation constant for the dimer/monomer equilibrium of MEK1 is 89 μM [17], the amount of dimer was 0.15 μM.

#### 2.3.1. Steady-State Intrinsic Fluorescence Measurements

The protein samples were excited at 280 and 295 nm in the pH range 2.0 to 12.0 to characterize a possible different behavior of tryptophan or tyrosine residues [26]. Chemical denaturations were also acquired by excitation at 280 and 295 nm. The slit widths were typically equal to 5 nm for excitation and emission in all cases. The fluorescence spectra were recorded between 300 and 400 nm. The signal was acquired and averaged for 1 s, and the increment of wavelength was set to 1 nm. Blank corrections were made in all spectra.

The chemical denaturations, either followed by fluorescence or far-UV CD, were carried out by dilution of the proper amount of either 7 M GdmCl or 8 M urea stock solutions and leaving the prepared samples incubating overnight at 5 °C. All the chemical denaturations were carried out at 50 mM (phosphate buffer), pH 8.1. Before the experiments were performed, the samples were left at 25 °C for 1 h. We have followed this protocol of unfolding denaturation equilibrations based on: (i) our previous experience with other large oligomeric proteins [27,28]; and (ii) a simple control fluorescent experiment where a spectrum acquired in the absence of denaturant and those acquired in the presence of either 6 M urea and 6 M GdmCl are compared, after 30 min of samples preparation, to see whether there are changes in the fluorescence intensity and/or in the position of the maximum wavelength. Chemical and pH denaturations followed by fluorescence were repeated twice, and, in both cases, the deviations between the two complete sets of denaturations were smaller than 10%.

In the pH-induced unfolding experiments, the pH was measured after completion of the experiments, and essentially no differences were observed with the pH values calculated from the buffer stock solutions. The pH was measured with an ultrathin Aldrich electrode in a Radiometer (Copenhagen, Denmark) pH-meter. The pH range explored was 2 to 12. The salts and acids used were as follows: pH 2.0–3.0, phosphoric acid; pH 3.0–4.0, formic acid; pH 4.0–5.5, acetic acid; pH 6.0–7.0, sodium dihydrogen phosphate; pH 7.5–9.0, Tris acid; pH 9.5–11.0, sodium carbonate; pH 11.5–13.0, sodium phosphate.

#### 2.3.2. ANS Binding

The excitation wavelength was 370 nm, and the fluorescence emission spectrum was measured from 400 to 600 nm. Slit widths were 5 nm for both excitation and emission. Stock solutions of ANS were prepared in water and diluted into the samples to yield a final concentration of 100 μM. The extrinsic fluorescence of the ANS probe was used to follow the chemical (urea or GdmCl) and pH denaturations. Protein concentrations in this case were also 4 μM (in protomer concentration). Signals from blank solutions were subtracted from the corresponding sample spectra in all cases.

#### 2.3.3. Thermal Denaturations

The average acquisition time of the experiments was 1 s with data collection every 0.2 °C. The heating rate was 60 °C/h. The ‘average time’ is the ‘sampling time’ of the instrument at each temperature. The MEK1 concentrations were in all cases 4 μM (in protomer concentration). Thermal scans were collected at 330 nm and 350 nm by excitation either at 280 or 295 nm through a temperature range from 25 to 85 °C. We chose these two wavelengths because the maximum wavelength of any solvent-exposed tryptophan is ~350 nm [26], and 330 nm is close to the maximum wavelength of the spectrum of native-like MEK1 (see Section 3.1.1) and is separated enough from 350 nm. The rest of the experimental set was the same as described above for the steady-state experiments. Thermal denaturations were not reversible at any pH value.

### 2.4. Far-UV Circular Dichroism Measurements (Far-UV CD)

Circular dichroism spectra were collected on a Jasco J810 spectropolarimeter (Hachioji, Tokyo, Japan) fitted with a thermostated cell holder and interfaced with a Neslab RTE-111 water bath. The instrument was periodically calibrated with (+)10-camphorsulphonic acid. A 0.1 cm-pathlength quartz cell (Hellma) was used in all cases. Either in the pH and chemical denaturations, spectra were corrected by subtracting the corresponding baseline.

#### 2.4.1. Steady-State Spectra

Isothermal wavelength spectra at different pH values were acquired at a scan rate of 50 nm/min with a response time of 2 s and averaged over six scans at 25 °C with a bandwidth of 1 nm and a step resolution of 0.2 nm. Far-UV measurements were performed in the same buffers used for the fluorescence experiments described above. The pH denaturations were repeated twice with new samples. In the two cases, the deviations were smaller than 10%.

In the chemical denaturation experiments, the far-UV CD spectra were acquired with the same experimental set as the pH denaturations. Experiments at different MEK1 concentrations (5 and 10 μM in protomer concentration) to test for urea denaturation were carried out at 5 °C. For the rest of the experiments, the amount of protein was the same as used in fluorescence (4 μM in protomer concentration).

#### 2.4.2. Thermal Denaturations

The experiments were performed with a response time of 8 s, a bandwidth of 1 nm and a step resolution of 0.2 °C. The heating rate was 60 °C/h, and the observed wavelength was 222 nm from 25 to 85 °C in 0.1 cm pathlength cells, with a total protein concentration of 4 μM. Buffers used were the same as those employed in the steady-state experiments. Scans aimed to test a possible drift of the signal of the spectropolarimeter did not show any difference. Thermal denaturations were not reversible at any pH for MEK1, as shown by: (i) the comparison of steady-state spectra acquired before and after the heating; and (ii) the sigmoidal changes in the voltage of the instrument [29].

### 2.5. Analysis of the pH, Thermal, and Chemical Denaturation Curves, and Stability Gibbs Energy Determination

The wavelength-averaged emission intensity, <λ>, in the fluorescence spectra was calculated as previously described [30]. The <λ> reports on changes in the spectral shape, as well as in the position; since it is an integral measurement, reflecting a global property of the full spectrum, it does have less error than measurements at a particular wavelength. The pH denaturations were analyzed assuming that both species, protonated and deprotonated, contributed to the fluorescence or CD spectra:

(1) $$X(pH)=\left(X_{a}\left(pH\right)+X_{b}(pH)\;10^{n\left(pH-pK_{a}\right)}\right)/\left(1+\;10^{n\left(pH-pK_{a}\right)}\right)$$

where *X* is the physical property being observed (raw ellipticity at a particular wavelength, <λ>, or fluorescence intensity at a particular wavelength); *X*_a_(pH) is the same physical property observed at acidic pH values; *X*_b_(pH) is the same physical property observed at basic pH values; p*K*_a_ is the apparent acid dissociation constant of the titrating group; and *n* is the Hill coefficient (which was close to 1 in all the curves reported in this work). The apparent p*K*_a_ reported was obtained from two measurements, prepared with different samples.

The thermal and chemical denaturation data for MEK1 were fitted to:

(2) $$X(x)=\left(X_{N}\left(x\right)+X_{D}(x)\;e^{\left(-∆G(x)/RT\right)}\right)/\left(1+\;e^{\left(-∆G(x)/RT\right)}\right)$$

where *R* is the gas constant, and *T* is the temperature (in K). The two values *X*_N_(*x*) and *X*_D_(*x*) correspond to the intrinsic values of the physical property described above, *X*(*x*), being monitored for the native and denatured protein, respectively, and *x* is either the temperature or the denaturant concentration. Both *X*_N_(*x*) and *X*_D_(*x*) were considered to be linear functions of the temperature or the denaturant concentration. Although urea and GdmCl denaturations of MEK1 were not reversible, in order to have a rather qualitative way to compare the different curves obtained by the various techniques used, the chemical denaturation curves were analyzed according to the linear extrapolation model [21,31], in which the Gibbs energy in Equation (2) is considered to depend linearly on the denaturant concentration: Δ*G*([D]) = *m*([D]_1/2_ − [D]), where [D] is the corresponding denaturant concentration (urea or GdmCl); [D]_1/2_ is the denaturant concentration at the midpoint of the transition; and *m* is the slope of the denaturant dependence of the Gibbs energy.

The thermal denaturations, either followed by intrinsic fluorescence or by far-UV CD, were irreversible as well. Nevertheless, we obtained an apparent thermal denaturation midpoint, *T*_m_, to allow for: (i) an estimate of the stability of MEK1, when possible; and (ii) a comparison with the DSC data. This value of *T*_m_ was obtained from Equation (2), where the stability Gibbs energy as a function of the temperature, ΔG, was given by:

(3) $$∆G(T)=∆H_{m}(1-\;T/T_{m})-\;∆C_{p}\;\left[\left(T_{m}-T\right)+T\;ln\left(T/T_{m}\right)\right]$$

where Δ*H*_m_ is the van’t Hoff unfolding enthalpy, and Δ*C*_p_ is the heat capacity change upon protein unfolding. The shape of Equations (2) and (3) does not impose restrictions on the value of the Δ*C*_p_ used in the fitting.

As MEK1 is a dimer (as shown in some of the resolved X-ray structures and our own DLS measurements, see below), when dimer dissociation occurred concomitantly with monomer unfolding, the equations providing the free-energy variation were corrected by a factor that takes into account the concentration-dependence of the second-order dissociation process as it has been previously described [32,33,34,35].

Fittings to Equations (1)–(3) by non-linear least-squares analyses were carried out by using KaleidaGraph version 3.5 (Synergy software, Reading, PA, USA).

### 2.6. Dynamic Light Scattering (DLS)

The DLS measurements were carried out in a Malvern Zetasizer Nano-ZS instrument (Malvern Panalytical Ltd., Enigma Business Park, Grovewood Road, Malvern WR14 1XZ; UK) at a fixed angle (Θ = 173°), equipped with a 10 mW helium-neon laser (λ = 632.8 nm) and a thermoelectric temperature controller, with the temperature fixed at 25 °C. Each experiment consisted of 5 measurements, with 15 runs and 10 s of acquisition time for each measurement. Samples at a concentration of 20 µM in protomer units (under these conditions, the amount of dimeric species is 2.5 µM, the dissociation constant is 89 µM [17]) were prepared in two different buffers: 0.5 M phosphate (pH 8.1) and 0.5 M acetic acid (pH 4.0), and measured in 3 mm-pathlength ultra-low-volume DLS cuvettes (ZEN2112, Hellma). Prior to analysis, samples were centrifuged for 10 min at 13,000 rpm using a benchtop centrifuge and filtered through a 0.1 µm pore-size polyethersulfone membrane to remove aggregates. Data were analyzed by using the Zetasizer software version 7.13 (Malvern Instruments Ltd., Enigma Business Park, Grovewood Road, Malvern WR14 1XZ; UK) to determine the hydrodynamic radius (*R*_h_) distributions.

The Z-average size was obtained by fitting the autocorrelation function with the cumulants method. The *R*_h_ of the protein in solution was calculated by applying the Stokes-Einstein equation.

### 2.7. Differential Scanning Calorimetry (DSC)

The average excess molar heat capacity of a solution of MEK1, with respect to a buffer solution, was measured as a function of temperature in an Auto-PEAQ-DSC instrument (MicroCal, Malvern-Panalytical, Enigma Business Park, Grovewood Road, Malvern WR14 1XZ; UK). Temperature scans were conducted between 10 and 95 °C, with a scan rate of 1 °C/min and no feedback gain, in phosphate buffer (50 mM, pH 8.1). The concentration of MEK1 was 4 μM, equal to the concentration employed in spectroscopic measurements; at that concentration, it was estimated that the fraction of monomer was around 0.93 (3.7 μM). Thus, most of the protein was in monomeric conformation, and the calorimetric signal-to-noise ratio was reasonably good. Additional unfolding experiments were performed in the presence of ATP (2 mM) in order to observe the stabilization induced by ATP binding.

Data were baseline-corrected and normalized by protein concentration. Because the thermal denaturations were irreversible, the data analysis consisted of a procedure in which the apparent unfolding temperatures and enthalpies were estimated. Briefly, because two transitions were observed for MEK1, the two-transition model was employed for the data analysis, which can be summarized in the following set of equations:

$$\begin{array}{c} \begin{array}{c} Q\left(T\right)=1+K_{1}\left(T\right)+K_{2}\left(T\right)+K_{1}\left(T\right)K_{2}\left(T\right)\\ K_{i}\left(T\right)=\;e^{\left(-{∆G}_{i}\left(T\right)/RT\right)} \end{array}\\ \begin{array}{c} {∆G}_{i}\left(T\right)={∆H}_{mi}\left(1-\frac{T}{T_{mi}}\right)+∆C_{Pi}\left(T-T_{mi}-T\ln\frac{T}{T_{mi}}\right)\\ {∆H}_{i}\left(T\right)={∆H}_{mi}+∆C_{Pi}\left(T-T_{mi}\right)\\ \begin{array}{c} \langle ∆H\rangle \left(T\right)=\frac{K_{1}\left(T\right){∆H}_{1}\left(T\right)+K_{2}\left(T\right){∆H}_{2}\left(T\right)+K_{1}\left(T\right)K_{2}\left(T\right)\left({{∆H}_{1}\left(T\right)+∆H}_{2}\left(T\right)\right)}{Q(T)}\\ \langle ∆C_{P}\rangle \left(T\right)=\frac{\partial \langle ∆H\rangle \left(T\right)}{\partial T} \end{array}\; \end{array} \end{array}$$

where *Q* is the partition function of the system; *K*_i_ is the equilibrium unfolding constant for each transition; *T*_mi_ is the unfolding temperature for each transition; Δ*G*_i_ is the unfolding Gibbs energy for each transition; Δ*H*_mi_ is the unfolding enthalpy for each transition; Δ*C*_Pi_ is the unfolding heat capacity for each transition; <Δ*H*> is the excess average molar unfolding enthalpy; and <Δ*C*_P_> is the excess average molar unfolding heat capacity (the observable provided by the calorimetric signal). Non-linear regression data analysis of the experimental data using Origin 7.0 (OriginLab, Northampton, MA, USA) allowed the estimation of the thermal unfolding parameters, basically unfolding temperatures, *T*_mi_, and unfolding enthalpies, Δ*H*_mi_.

### 2.8. Isothermal Titration Calorimetry (ITC)

The interaction of MEK1 with ATP was assessed in a PEAQ-ITC instrument (MicroCal, Malvern-Panalytical, Enigma Business Park, Grovewood Road, Malvern WR14 1XZ; UK). An ATP solution (200 μM) was titrated into a MEK1 solution (4 μM, in protomer units) at 25 °C in phosphate buffer (50 mM, pH 8.1), by programming a series of nineteen 2-μL injections with a spacing of 150 s, a reference power of 10 μcal/s, and a stirring speed of 750 rpm. As stated above, the concentration of MEK1 was 4 μM (in protomer units), equal to the concentration employed in spectroscopic and DSC measurements, accomplishing a balance between sufficient calorimetric signal and predominant monomeric protein population.

After baseline-correcting the thermogram, the binding isotherm was generated by integrating the heat effects per injection and normalizing by the amount (number of moles) of ligand (ATP) injected. Nonlinear least-squares regression data analysis considering a model with a single ligand binding site and using a user-defined fitting routine defined in Origin 7.0 (OriginLab, Northampton, MA, USA) provided estimates of the interaction parameters: association constant *K*_a_, interaction enthalpy, Δ*H*, and apparent stoichiometry, *n*.

### 2.9. Simulated Thermal Unfolding

The unfolding of MEK1 was simulated by using the computational technique known as CNA [20]. This methodology, based on graph theory applied to a protein inner constraint network, can reveal the presence of distinct transitions along the unfolding pathway. The unfolding is modeled by progressively removing the constraints that form the network of non-bonded interactions in the native protein, simulating a gradual loss of structural rigidity.

The starting model of the monomer of MEK1 was retrieved from the AlphaFold database [36]. The use of an AlphaFold model, in spite of a large number of X-ray structures available for this protein and differing from each other by a few structural details, was preferred precisely to avoid any bias and choosing one specific structure over any other. In fact, because of the presence of such a large variety of almost equivalent structures corresponding to the MEK1 sequence in the PDB, the AlphaFold structure (which included all of them in the training dataset) is equivalent to a highly reliable average conformation of the protein. The structure of the dimer was built by using AlphaFold-Multimer on ColabFold [37]. Both protein species were protonated by using UCSF Chimera [38]. These structures were given as input to the CNAnalysis web interface [39], choosing the single-network/single-structure analysis option with default simulation parameters. Conformational transitions were automatically identified by the CNA algorithm as sudden changes (from rigid to flexible) in the protein connectivity network, along the folding process monitored at increasing energy.

The CNA technique was also used to identify the unfolding nuclei in the protein structure, which are the weak spots in the protein structure where the unfolding begins and, therefore, correspond to polypeptide regions prone to start the denaturation process of the whole protein. This is best captured by using an ensemble of networks from the single protein structure provided as input, introducing ‘fuzzy’ non-covalent constraints that randomly break/form in the native monomer [40]. To this end, we chose the ensemble-of-networks/single-structure analysis option, with default simulation parameters.

## 3. Results

### 3.1. MEK1 Acquired a Native-like Conformation in a Narrow pH Range

To describe and measure the conformational features of MEK1 and its conformational stability, we ought to determine the pH interval in which the protein acquired a native-like structure. To that goal, we used several complementary biophysical and spectroscopic approaches. These techniques were: intrinsic fluorescence (i.e., that provided by the tyrosine (9) and tryptophans (2) in the protein monomer of the protein); 8-anilino-1-sulfonic-acid (ANS) fluorescence; far-UV circular dichroism (CD); and dynamic light scattering (DLS). These techniques together provide complementary information on distinct structural features of the protein. In particular, we used intrinsic fluorescence to follow changes in the tertiary structure of the protein in the proximity of tyrosine and tryptophan residues. We used ANS fluorescence to display the burial of solvent-exposed hydrophobic patches and to detect the presence of possible partially folded species [41]. We carried out far-UV CD experiments to detect secondary structure changes. And, finally, we used DLS to determine the hydrodynamic size of the polypeptide chain under different conditions.

**Figure 2:**
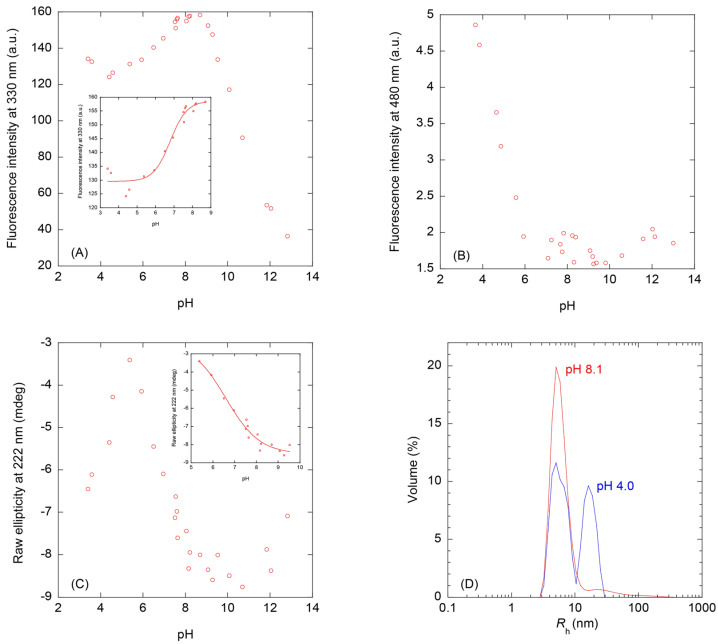
pH-induced structural changes in MEK1 followed by spectroscopic and biophysical techniques. (**A**) Variations in the intrinsic fluorescence spectra of MEK1 monitored by the changes in fluorescence intensity after excitation at 280 nm. Inset: The red line is the fitting to Equation (1) of the protonation/deprotonation transition observed. (**B**) Changes in the fluorescence intensity of ANS (at 100 μM) were followed at 480 nm. (**C**) Changes in raw ellipticity at 222 nm from the far-UV CD spectra. Inset: The red line is the fitting of the protonation/deprotonation transition observed in Equation (1). The pH denaturations followed by fluorescence (intrinsic or ANS) and CD were repeated twice, and, in both cases, the deviations between the two complete sets of denaturations were smaller than 10%. (**D**) Volume distribution profiles at pH 8.1 and 4.0 from the DLS experiments (at 20 μM in protomer units of MEK1). All the experiments were performed at 25 °C.

In all these techniques, we defined the native-like conformation adopted by the protein, when a plateau for the monitored experimental parameter (the $X\left(pH\right)$ in Equation (1)) was reached. For MEK1, this occurred in intrinsic fluorescence experiments when the intensity reached the largest value (Figure 2A); in ANS fluorescence experiments, when the intensity reached the smaller value (Figure 2B); in far-UV CD experiments when the ellipticity reached the largest (in absolute terms) value (Figure 2C); and in DLS experiments when there mainly a single species, with the proper size similar to that obtained from the X-ray structure [17] (Figure 2D).

#### 3.1.1. Intrinsic Steady-State Fluorescence and Thermal Denaturations Followed by Intrinsic Fluorescence

The fluorescence spectrum of MEK1 showed a wavelength corresponding to the maximal intensity at ~340 nm, and the position of this maximum remained unchanged at acidic and physiological pH values (Figure S1A,B), indicating that the two tryptophans were buried during any transition (as the maximum wavelength in the case of any solvent-exposed tryptophan is ~350 nm [26]). We tried to measure the solvent-accessibility of tryptophans and tyrosines at low and physiological pH values by using KI quenching, but at acidic pH values, the sample precipitated, and then, no conclusive results were obtained. Probably this was due to the presence of species with a high amount of solvent-exposed hydrophobic patches (see below in this same section for the ANS results) and to the presence of partially folded self-associated species (see DLS results, Section 3.1.4). It is well-known that partially folded species with a large amount of solvent-exposed hydrophobic patches have a high tendency to precipitate at high ionic strength, as that used in KI quenching experiments [41]. Conversely, at basic pH values, the emission spectral intensity was not only lower, but there was also a shift in the wavelength maximum towards 350 nm, indicating that at high pH values, at least one out of two tryptophans of the monomer became solvent-exposed.

The fluorescence intensity, either after excitation at 280 or 295 nm, was modified as the pH changed, resulting in a bell-shaped variation with two transitions: one close to acidic/physiological pH values, and another at basic ones (Figure 2). We could not determine the p*K*_a_ of the basic transition due to the absence of a baseline at high pH values, but this transition, which caused a change in the maximum wavelength (see above), might be due to titration of at least one of the nine tyrosine residues in each monomer. The transition occurring at acidic pH values had an apparent p*K*_a_ value of 6.9 ± 0.8 (with a Hill coefficient value, *n*, of 0.8, Figure 2A, inset). The plateau of the bell-shaped curve comprised the pH range between 8.0 and 9.0. The same behavior was observed by following the variation of <λ>, although the amplitude of the transition occurring at acidic/physiological pH values was smaller.

Thermal denaturations performed at several pH values (pH 5.4, 8.1 and 12.1) were carried out by following the changes in the intrinsic fluorescence in MEK1 at several wavelengths (typically 330 and 350 nm) after excitation at 280 or 295 nm. At any of these pH values, we could not detect any sigmoidal transition, and scattering of the fluorescence signal was observed at high temperatures (Figure S1C). The lack of an observed sigmoidal transition, especially at pH 8.1, was probably due to thermo-quenching.

#### 3.1.2. ANS-Binding Fluorescence

At low pH, the ANS fluorescence intensity at 480 nm was larger and decreased as the pH was raised (Figure 2B), suggesting that, under acidic conditions, MEK1 showed solvent-exposed hydrophobic regions [41]. However, we could not determine its protonation/deprotonation midpoint because of the lack of an acidic baseline. From the shape of the curve, we can conclude that this protonation/deprotonation was not the same as that monitored by intrinsic fluorescence after excitation at 280 or 295 nm (Figure 2A), indicating that the acquisition of native-like fluorescence occurred at more basic pH values than the burial of solvent-exposed hydrophobic patches.

On the other hand, and conversely to what happened for the intrinsic fluorescence, we did not observe any protonation/deprotonation at high pH values, indicating that the possible changes in the protonation of tyrosines did not lead to the development of a large amount of nearby solvent-exposed hydrophobic patches.

#### 3.1.3. Far UV CD

The CD spectrum of MEK1 at pH 7.0 (Figure S2) showed two minima at around 208 and 222 nm, suggesting the presence of a considerable fraction of α-helix, as also shown by the X-ray structure of the protein [17]. The spectrum at acidic pH values was different from that at pH 7.0, and it had a smaller ellipticity value (Figure S2A). In general, the spectra had a lower ellipticity value at pH < 6.0, indicating that at acidic pH values, the domain had lost some of its secondary structure. This loss leads to the solvent exposure of a large amount of nearby hydrophobic residues (as suggested from the experiments in the presence of ANS, Figure 2B). The changes in ellipticity at 222 nm (Figure 2C) had two transitions. We could not determine the p*K*_a_ of the most acidic one, whereas the p*K*_a_ of the other was 6.9 ± 0.9 (obtained from the experimental values of the ellipticity at 222 nm measured between pH 5.5 and 9.5, and with an *n* value of 0.9, Figure 2C, inset). This value was similar, within the error, to the p*K*_a_ value observed from the variation in the intrinsic fluorescence (Figure 2A inset); these findings suggest that the acquisition of native-like tertiary structure paralleled the acquisition of native-like secondary structure and occurred at similar pH values. On the other hand, we could not obtain any reliable value for a p*K*_a_ at the transition observed at very acidic pH values (i.e., at pH < 5.5) due to the absence of the acidic baseline. At basic pH values, we also observed a decrease (Figure 2C) in the raw ellipticity.

Then, from the behavior of the ellipticity at 222 nm in the far-UV CD spectra, we conclude that the protein acquired a native-like secondary structure in the range between pH 8.0 and 9.0.

In contrast to what happened in the thermal denaturations followed by fluorescence, the thermal denaturations followed by far-UV CD showed a single sigmoidal transition at pH 8.1, whereas at both lower and higher pH values of 5.4 and 12.1, respectively, we observed a linear decrease in the ellipticity at 222 nm as the temperature was raised (Figure S2B). The thermal denaturation at pH 8.1 was irreversible, as judged from: (i) the changes in the voltage of photomultiplier [29]; and (ii) the presence of precipitation in the cell after the heating; however, we could obtain an apparent thermal denaturation midpoint from the thermal denaturation (Equations (2) and (3)) of 325 ± 5 K (52 ± 5 °C).

#### 3.1.4. DLS

We carried out DLS measurements at two pH values where, from the intrinsic fluorescence, ANS fluorescence and far-UV CD results, we could conclude that the protein had a native-like conformation (pH 8.1) or alternatively, it had not acquired a native-like one (pH 4.0).

At pH 8.1, MEK1 had acquired a native-like conformation, and the DLS yielded two peaks with *R*_h_ values of 5.7 ± 1.4 nm (which accounts for 94% of total volume) and 30 ± 40 nm (which is 6% of total volume), based on the Stokes-Einstein equation (Figure 2D), which assumes an approximately spherical shape. Calculations from the empirical relationship between a polypeptide length and its hydrodynamic radius (*R*_h_ = (4.75 ± 1.11) *N* ^0.29 ± 0.02^, where *N* is the number or residues in MEK1 [42]) led to values of 2.6 ± 0.6 nm (for the monomer) and 3.3 ± 0.7 (for the dimer), which could explain the major peak observed in the DLS results at pH 8.1. As the theoretical hydrodynamic radii of both species were very similar, it was difficult to distinguish between the two populations in DLS measurements; hence, the main peak included both conformations (at 20 µM, where a ~12.5% of the population was in the dimeric form, taking into account a dissociation constant of 89 μM [17]). The differences between the DLS estimations and the theoretical results could be due to: (i) the fact that the estimation of the *R*_h_ from DLS measurements assumes the Stokes-Einstein equation and the shape of the dimeric MEK1 species is elongated (Protein Data Bank (PDB) ID: 1S9J [17,22,23]); and (ii) the empirical nature of the equation used. The peak leading to the minor fraction indicated that the protein might exist in higher-order oligomers, which would explain why the peak width, i.e., the dispersion, was so high.

However, at pH 4.0, where the protein had not acquired a native-like tertiary structure (Figure 2A), nor a secondary-like one (Figure 2C) and had solvent-exposed hydrophobic patches (Figure 2B), the proportion of species (monomer, dimer, or possibly higher-order oligomers) changed (Figure 2D). Two clearly distinct peaks were observed. The peak with the highest volume percentage (63% of total volume) corresponded to a size of 5.8 ± 1.5 nm, which included both monomer and dimer species, with no possibility of separating them, as mentioned before, on the basis of the data collected at pH 8.1. In contrast to what happened at pH 8.1, 30% of these species were now in the oligomeric state, accounting for 37% of the total volume and having a size of 17 ± 4 nm. This species could be explained as due to the presence of non-native, high-order, aggregated products, probably partly folded, resulting from self-associated species (because of the presence of the solvent-exposed patches in each polypeptide chain of MEK1, as shown by the intense fluorescence of the ANS (Figure 2B)). It is interesting to note that we also calculated the theoretical *R*_h_ for MEK1 in a completely unfolded state (*R*_h_ = (2.21 ± 1.07) *N* ^0.57 ± 0.02^ [42]), but such a value did not agree with that estimated at the acidic pH. Then, taken together, these DLS results confirm the absence of native-like structure at acidic pH values and the presence of species with a high tendency to self-associate (probably due to the large amount of solvent-exposed hydrophobic patches).

To sum up, all the biophysical and spectroscopic probes suggest that MEK1 acquired the native-like tertiary and secondary structures concomitantly around pH 8.0, with this conformation remaining stable between pH 8.0 and 9.0. At acidic pH values, the burial of solvent-exposed hydrophobic patches occurred before the concomitant consolidation of the secondary and tertiary structures. In contrast, at basic pH values, the protonation/deprotonation of tyrosine residues seemed to cause concomitant changes in the native-like tertiary and secondary structures.

### 3.2. Conformational Stability of MEK1 at pH 8.1

We tried to measure the conformational stability of MEK1 at pH 8.1, where the protein had acquired native-like secondary and tertiary structures, by calorimetric denaturations followed by DSC and chemical denaturations monitored by using fluorescence (in particular, both intrinsic and ANS fluorescence) and far-UV CD. Denaturations using both chaotropic agents were irreversible, and, therefore, the unfolding parameters must be considered as apparent parameters and as a qualitative guide to discuss our findings.

#### 3.2.1. Chemical (Urea and GdmCl) Denaturations of MEK1

**Figure 3:**
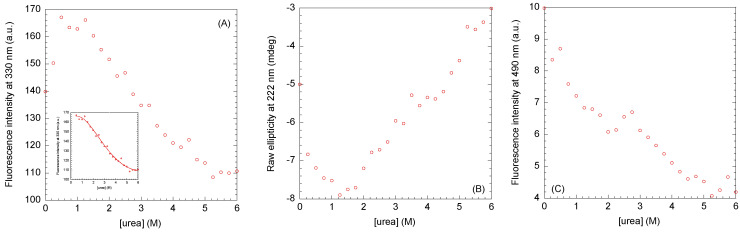
Urea denaturation of MEK1 followed by spectroscopic techniques. (**A**) Variations in the intrinsic fluorescence intensity after excitation at 280 nm. Inset: The red line is the fitting to Equation (2) of the second transition observed. (**B**) Variations in raw ellipticity at 222 nm. (**C**) Variations in ANS fluorescence intensity after excitation at 370 nm. Experiments were performed at 25 °C with a protomer concentration of 4 μM. The urea denaturations, followed by intrinsic and ANS fluorescence and CD, were repeated twice and, in both cases, the deviations between the two complete sets of denaturations were smaller than 10%.

**Figure 4:**
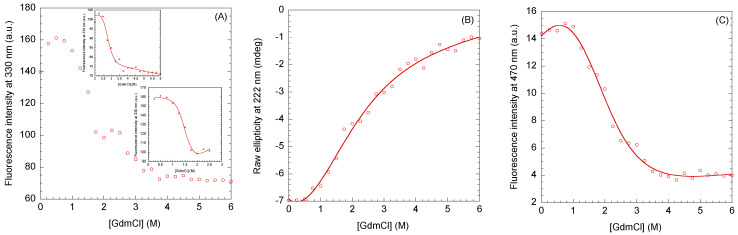
GdmCl denaturation of MEK1 followed by spectroscopic techniques. (**A**) Variations in the intrinsic fluorescence intensity after excitation at 280 nm. Insets: Fittings of the two transitions observed by following the intrinsic fluorescence experiments. (**B**) Variations in raw ellipticity at 222 nm. (**C**) Variations in ANS fluorescence intensity after excitation at 370 nm. The red lines in panels (**B**,**C**), and in the two insets of panel (**A**) are the fittings to a two-state equation (Equation (2)) with the linear extrapolation model. Experiments were performed at 25 °C with a protomer concentration of 4 μM. The GdmCl denaturations, followed by intrinsic and ANS fluorescence and CD, were repeated twice and, in both cases, the deviations between the two complete sets of denaturations were smaller than 10%.

(a) Conformational changes in MEK1 at pH 8.1 with urea: We carried out chemical denaturation experiments in the presence of urea by using (intrinsic and ANS) fluorescence and CD. At room temperature, and at 4 μM (in protomer units) of MEK1, the intrinsic fluorescence intensity, either by excitation at 280 or 295 nm, showed two transitions at any wavelength: (i) one occurring between 0 and 0.75 M urea; and (ii) the second one at higher urea concentration (Figure 3A). Due to the absence of a baseline in the first one, we could not obtain an apparent midpoint for the transition, [urea]_1/2_; however, for the second one (Figure 3A, inset), we obtained [urea]_1/2_ = 1.8 ± 1.0 M and *m* = 660 ± 136 cal mol^−1^ M^−1^. On the other hand, the variation of <λ> (either after excitation at 280 or 295 nm) yielded a single transition with [urea]_1/2_ = 3 ± 1 M and *m* = 390 ± 230 cal mol^−1^ M^−1^ (Figure S3A).
  Furthermore, the results from the CD data indicated, as well, a first transition between 0 and 1 M urea and a second one between 1 and 6 M urea (Figure 3B). We could not fit any of the transitions because of the lack of a native baseline for the first transition and of an unfolding baseline for the second. Moreover, the urea-denaturation followed by ANS also showed two transitions (Figure 3C): (i) the first one occurring between 0 and 2 M urea; and (ii) the second one occurring between 2 and 6 M urea. Unfortunately, attempts to fit this second transition yielded unreliable results.
  We suspected that the transition observed at low urea concentrations was probably due to dimer dissociation, or alternatively, unfolding of one of the two lobes of the protein. To test that hypothesis, and since we could acquire experiments below room temperature only in the spectropolarimeter, we designed a urea denaturation experiment at low concentration (5 μM) and high (10 μM) protomer concentration at low temperature (5 °C). We did not use GdmCl as a denaturing agent because of the absence of the first transition in the far-UV CD experiments (see below in this same section). If that first transition was due to dimer dissociation, we should see at low concentration and low temperature the same transition observed at high temperature (Figure 3B), but such a transition should move towards higher concentrations of denaturant due to Le Châtelier principle, i.e., a concentration-dependent midpoint transition, [D]1/2 [32,33,34,35]. When we carried out the experiments, the first transition occurring at low urea concentrations was clearly observed in the diluted sample (Figure S4) together with the transition observed at the highest urea concentrations; however, at the high protein concentration, we only observed a single transition occurring at high urea concentrations, probably due to the concomitant occurrence of dimer dissociation and unfolding of the monomer.
  Then, taken together, and due to the variety of behaviors observed among the different probes during the denaturation, our findings suggest that unfolding of MEK1 caused by the presence of urea: (i) was irreversible; and (ii) it was not a two-state transition, involving conformational intermediate states, pointing to a low (un)folding cooperativity [43]; Unfortunately, the irreversibility of the denaturation process precluded to determine a thermodynamic free energy of unfolding.
(b) Conformational changes in MEK1 at pH 8.1 with GdmCl: Experiments with GdmCl by monitoring the intrinsic fluorescence intensity showed three transitions: (i) one at the same range of concentrations observed with urea (i.e., between 0 and 0.75 M of GdmCl); (ii) another one between 0.75 and 2 M of GdmCl; and (iii) the final one between 2 and 6 M of GdmCl (Figure 4A). In this case, we could fit the second and last transitions to consecutive sigmoidal curves. In the second transition, we obtained [GdmCl]_1/2_ = 1.52 ± 0.08 M and *m* = 3 ± 1 kcal mol^−1^ M^−1^; and the third one yielded: [GdmCl]_1/2_ = 2.4 ± 0.2 M and *m* = 2.4 ± 0.4 kcal mol^−1^ M^−1^ (Figure 4A inset). On the other hand, the variation of <λ> (either after excitation at 280 or 295 nm) yielded a single transition with [GdmCl]_1/2_ = 1.9 ± 0.3 M and *m* = 760 ± 112 cal mol^−1^ M^−1^ (Figure S3B).
  Furthermore, the denaturation followed by far-UV CD also yielded a single, low-cooperative (i.e., small m-value) transition, with [GdmCl]_1/2_ = 2.8 ± 0.2 M and m = 626 ± 245 cal mol^−1^ M^−1^ (Figure 4B). Attempts to fit the far-UV CD data to a curve with more than one transition did not lead to reliable results (as judged from the F-test and the χ2 values of the fittings). The chemical denaturation in the presence of ANS also yielded a single transition with [GdmCl]_1/2_ = 1.6 ± 0.3 M and m = 930 ± 160 cal mol^−1^ M^−1^ (Figure 4C).
  To sum up, these results indicated that the unfolding of MEK1 in the presence of GdmCl: (i) was irreversible; and (ii) was not a two-state transition, as concluded from the different m- and [GdmCl]_1/2_—values obtained by using distinct probes and biophysical techniques [43] but rather it involved several intermediates (as concluded from the distinct [GdmCl]_1/2_ and m-values obtained by the different techniques).

#### 3.2.2. Heat Denaturation of MEK1 in the Absence and in the Presence of ATP Followed by DSC at pH 8.1

**Figure 5:**
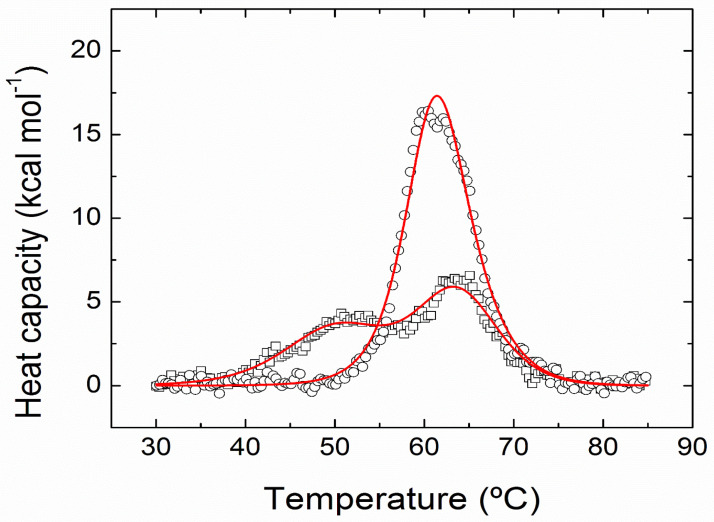
Differential scanning calorimetry of MEK1 in the absence and in the presence of ATP. The thermogram corresponding to the thermal unfolding of isolated MEK1 (blank squares) was analyzed with a two-transition model in order to estimate the apparent stability parameters (red line), although the protein unfolding was irreversible. The thermogram corresponding to the thermal unfolding of MEK1 in the presence of 2 mM ATP (blank circles) was also analyzed with a two-transition model (red line).

According to the thermal denaturation monitored by DSC, the unfolding of isolated MEK1 revealed two sequential unfolding transitions with apparent unfolding temperatures (*T*_m_) of 52.9 and 63.4 °C, with a global apparent unfolding enthalpy of 116 kcal mol^−1^ (Figure 5, blank squares). A more detailed analysis employing a model considering two unfolding transitions provided refined estimations of the unfolding temperatures (*T*_m1_ = 49.2 °C and *T*_m2_ = 62.6 °C) and the unfolding enthalpies (Δ*H*_m1_ = 53 kcal mol^−1^ and Δ*H*_m2_ = 70 kcal mol^−1^). This indicates that there was no full cooperativity and the protein contained two independent and energetically distinguishable domains regarding its folding behavior. These two energetic domains might correspond to the two protein lobes: the smaller N-terminal lobe (residues 1–145), which includes the first ~20 disordered residues, and the larger C-terminal one (residues 146–393).

In the presence of ATP, a single unfolding was observed, with an apparent *T*_m_ of 61.1 °C and an overall unfolding enthalpy of 163 kcal mol^−1^ (Figure 5, blank circles). A detailed data analysis applying the model considering two unfolding transitions, provided refined estimations of the unfolding temperatures (*T*_m1_ = 60.9 °C and *T*_m2_ = 62.7 °C) and the unfolding enthalpies (Δ*H*_m1_ = 105 kcal mol^−1^ and Δ*H*_m2_ = 68 kcal mol^−1^). These results indicate that the low-stability unfolding domain was stabilized by the binding of ATP, while the high-stability domain remained unaffected by ATP binding, and the two unfolding transitions overlapped, resulting in an apparent single unfolding transition. The interaction with ATP increased the overall structural stability and the unfolding cooperativity of MEK1.

#### 3.2.3. Interaction of MEK1 with ATP Monitored by ITC at pH 8.1

**Figure 6:**
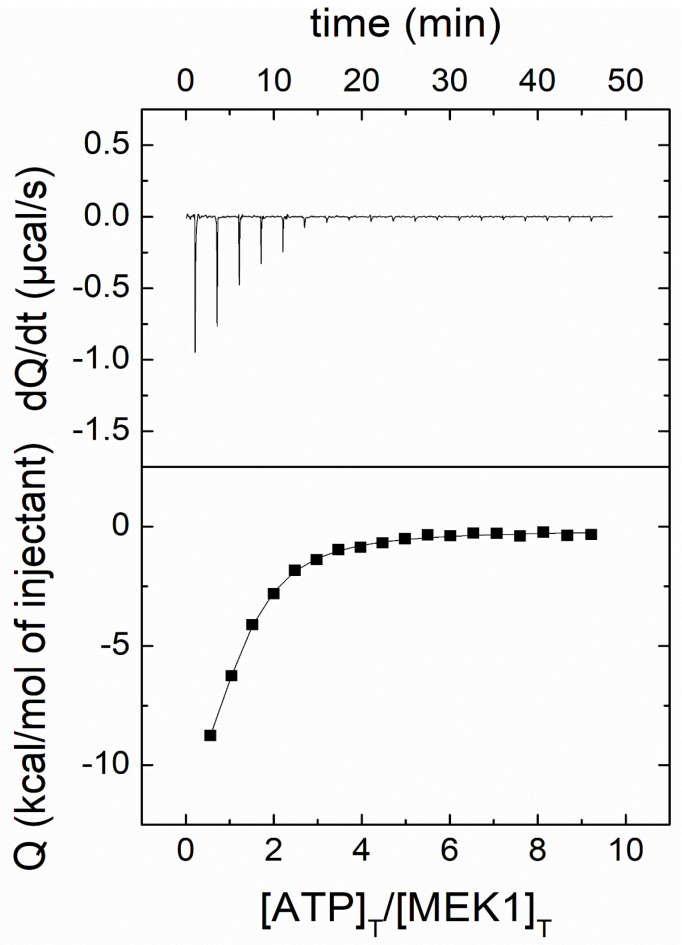
Quantitative determination of the binding of MEK1 to ATP as monitored by ITC. Calorimetric titration of MEK1 with ATP. The upper panel shows the thermogram (thermal power as a function of time), and the lower panel shows the binding isotherms (ligand-normalized heat effects per injection as a function of the molar ratio in the calorimetric cell). The continuous curve corresponds to the non-linear least-squares fitting according to a single ligand-binding site interaction model. Experiments were performed at 25 °C.

According to the calorimetric titration, MEK1 interacted with ATP with an association constant of 5.1 × 10^5^ M^−1^, corresponding to a dissociation constant of 2 μM. The binding was exothermic and enthalpically driven, with an observed binding enthalpy of −15.3 kcal mol^−1^ (Figure 6). This moderate binding affinity was responsible, at least in part, for the stabilization effect observed in the DSC experiments.

### 3.3. In Silico MEK1 Denaturations

Thermal and chemical unfolding of MEK1 appeared to be a complex process with a hierarchy of possible intermediates (Section 3.2). For this reason, we applied CNA [39], a computational technique capable of revealing the presence of transitions along the denaturation pathway, not only for proteins with a complex architecture but also for small folded domains [44]. The unfolding was observed at increasing energy E, with transitions corresponding to an abrupt loss of structural rigidity while gradually removing non-bonded constraints in the network of native protein interactions.

**Figure 7:**
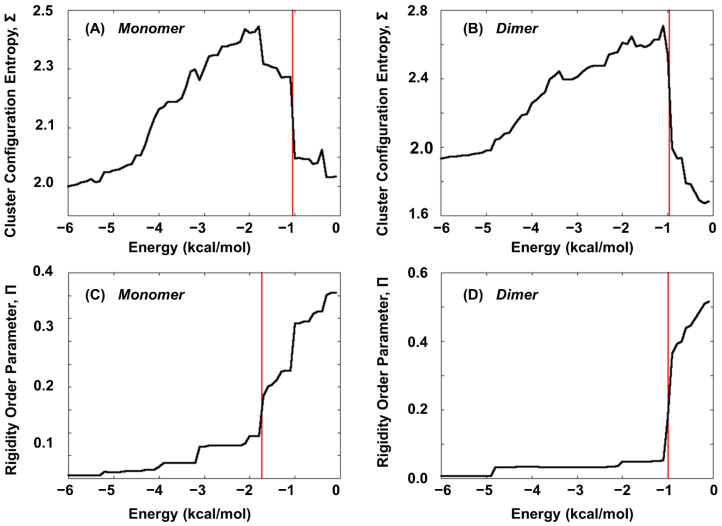
Global flexibility index of MEK1 simulated by CNA as a function of the unfolding reaction coordinate. (**A**) Cluster conformation entropy, Σ, measuring the disorder of the network of non-bonded interactions for the monomeric species. (**B**) Cluster conformation entropy of MEK1 dimer. (**C**) Rigidity order parameter, Π, describing the number of constraints in the network for the monomeric species. (**D**) Rigidity order parameter of MEK1 dimer. The unfolding reactions progress at decreasing energy (i.e., from right to left along the *x*-axis). The red lines indicate main transitions identified by the CNA algorithm.

Figure 7 shows the unfolding process as monitored by the cluster configuration entropy, Σ, and the rigidity order parameter, Π. Both Σ and Π are plotted as a function of the folding/unfolding reaction coordinate represented by E, with the unfolding process progressing from right to left along the *x*-axis. All the curves showed a first transition at −1 kcal mol^−1^, which can be observed for both the monomeric and dimeric species of MEK1. This transition corresponded to the melting of the most labile of the two unfolding domains (the initial steps of this process may also trigger the dissociation of the dimer). A second transition could be observed for the monomeric protein species at −1.72 kcal mol^−1^. This transition is indicative of the melting of the high-stability unfolding domain of MEK1, which completes the whole protein denaturation. After such an event, the cluster configuration entropy reached its maximum and then gradually collapsed while residual protein structures disappeared.

It is interesting to note that the reaction coordinate E can be converted to an approximate temperature scale [45]. The observed transitions during protein unfolding generally depend on the protein size and fold; therefore, the values obtained in the simulations may not correspond accurately to the experimental unfolding temperatures observed in wet-laboratory experiments. Nevertheless, the temperatures found in such transitions for MEK1 (48 and 61 °C, respectively) were very close to the two transitions measured by using DSC (49.2 and 62.6 °C) (Figure 5), and very close to the apparent thermal denaturation of the transition measured by far-UV CD (Figure S2) (52 °C; Section 3.1.3).

Our simulation data suggest that the high-stability domain (i.e., that with the largest thermal denaturation midpoint as measured by CD) was the larger one (C-terminal lobe), and the low-stability domain was the smaller one (N-terminal lobe). Upon addition of ATP, ATP is bound at the interface of the two lobes, and ATP binding affects the stability of the N-terminal lobe and brings it to the same stability as the C-terminal, and the latter “becomes the overall stability of the whole protein”.

**Figure 8:**
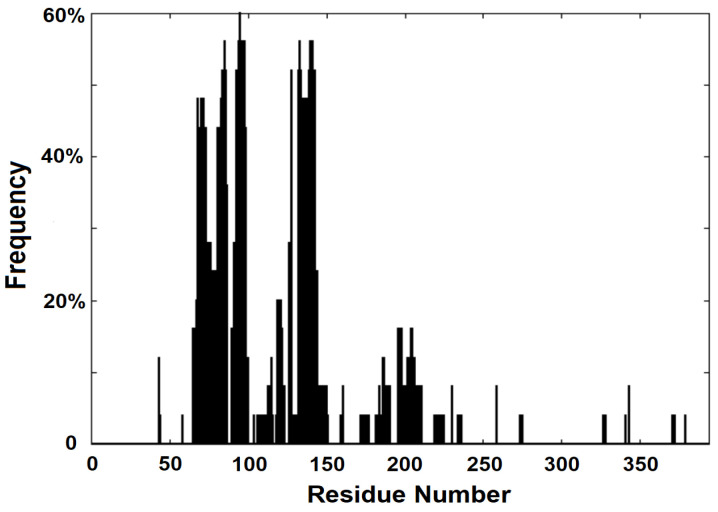
Unfolding nuclei in the sequence of MEK1 simulated by CNA as a function of protein residue. Regions with a higher frequency indicate weak spots in the protein structure where the unfolding is more likely to occur. The analysis was performed by using the option ensemble-of-networks on the single structure of the MEK1 monomer.

Finally, we used CNA (in ensemble-of-networks mode) also to identify the unfolding nuclei, which are the weak spots in the protein structure where the unfolding starts. The results reported in Figure 8 show that higher frequencies (i.e., stronger hot spots) are found for protein residues 55–145. This finding demonstrates that the most labile of the two unfolding domains is the N-terminal lobe of MEK1 (encompassing residues 1–145). Conversely, the protein domain with higher stability is the C-terminal lobe (residues 146–393), which is comparatively larger and thus should be the one with a higher melting temperature in the DSC experiments in the absence of ATP (Figure 5, blank squares).

## 4. Discussion

MEK1 is phosphorylated by the upstream Raf kinase at two serine positions, and it must phosphorylate the downstream kinase ERK. In addition, it must allocate the ATP molecule necessary for such phosphorylation [17,22,23]. Therefore, MEK1 must have an intrinsic high flexibility, which allows the interaction with different partners. Whereas the structural determinants of such flexibility might be well-known, less is known about the dynamics and stability of MEK1. In particular, the conformational features of MEK1 under the most common unfolding conditions (pH, temperature, and the presence of chemical denaturants) have been little explored so far.

The first conclusion of our work is that the acquisition of the native-like tertiary and secondary structures, and the burial of solvent-exposed hydrophobic patches—monitored by fluorescence, far-UV CD and ANS fluorescence, respectively—as the pH was varied, did not occur in a concomitant manner (Figure 2). Thus, upon transitioning from acidic pH values, first the burial of solvent-exposed hydrophobic patches occurred at a very acidic pH value (<5.0), and then the acquisition of native-like secondary and tertiary structures happened (with a p*K*_a_ ~6.0). At acidic pH, and although we could not determine the exact p*K*_a_ of the transition, the loss of native-like structures could be associated with the protonation/deprotonation of aspartic or glutamic acid residues. On the other hand, the transition observed by far-UV CD and intrinsic fluorescence with a p*K*_a_ ~6.0 could be due to one or more titrating histidine residue(s) [46,47,48]—as MEK1 contains nine histidines—although we cannot unambiguously rule out that this effect could be due to an aspartic or glutamic acid with an unusually high p*K*_a_ value.

The findings that the populated species at low pH showed a large ANS fluorescence intensity and altered CD signals (with lower ellipticity, in absolute value, than the species populated at pH 8.1) suggest that they are non-native partially folded species [41], as we could further confirm by the absence of thermal unfolding sigmoidal curves followed by fluorescence or CD at low pH values (Figures S1 and S2); furthermore, the species at low pH values also showed a high tendency to aggregate as suggested by both the DLS (Figure 2D) and the ANS (Figure 2B) results. Our findings suggest a high sensitivity to environmental cues, reflecting an extensive propensity to conformational rearrangements favoring the interaction with multiple biological partners. It is important to indicate that, although we cannot provide atomic details on the particular polypeptide patches involved in those transitions, our results indicate that there are distinct regions in the protein that are suffering conformational changes. It could be thought that, apart from the molecular simulations used in our work, NMR could provide some clues on those polypeptide patches; however, (i) the fact that the protein has a large amount of solvent-exposed hydrophobic patches; and (ii) the high protein concentrations needed in the NMR experiments suggest that trying to obtain atomic-detail information would result in protein precipitation or NMR spectra with a large signal broadening, hampering assignment.

The thermal unfolding monitored by DSC revealed two transitions. The transition with the lower unfolding temperature showed an unfolding temperature *T*_m_ of 49.2 °C, similar to that observed in the thermal unfolding followed by far-UV CD (Figure S2). The presence of two sequential transitions in the DSC experiments indicates that there was no complete folding cooperativity, which represents additional evidence for the conformational plasticity of MEK1. The stability and the folding cooperativity were enhanced by ATP binding in between the two protein lobes, which helps to stabilize the region in MEK1 with the lowest stability, but even in the presence of ATP, the transitions, as indicated previously, were irreversible.

The estimated binding affinity for MEK1 interaction with ATP was moderate, with a dissociation constant of 2 μM. This agrees with a previously observed dissociation constant of 3 μM for ATP and other analogs interacting with the inactive, unphosphorylated MEK1, and the considerable binding affinity even in the absence of magnesium ions [49,50].

The simulation results obtained by using the CNA technique indicate that the two unfolding temperatures obtained by DSC corresponded to the dissimilar thermal behavior of the two protein lobes: a more labile N-terminal region and a C-terminal lobe with higher stability. Together with the stepwise behavior of the curve of the rigidity order parameter (Figure 7A), these findings suggest a high conformational plasticity of MEK1 in spite of its apparent structural simplicity. The observation that CNA could capture the hierarchy of folding events also suggests that the topology of the network of non-bonded interactions within the protein structure played an important role in the stability of MEK1. The binding of ATP contributed to stabilizing the structure of MEK1 by bridging the two protein lobes, forming an additional network of interactions that may add an important contribution to the moderate binding affinity of ATP for this protein (Figure 6).

## 5. Conclusions

MEK1 is a dual-specificity kinase that plays a critical role in the ERK-MAPK pathway. It participates in signal transduction in the cells, regulating their survival, differentiation, and proliferation. As a consequence, it is involved in several fundamental physiological processes, and it is considered a pharmacological target for cancer therapy. In this work, we characterized the stability of MEK1 with respect to changes in pH and temperature, as well as due to chemical denaturants, by using several different biophysical techniques and with the help of computational modeling. The presence of a hierarchy in the unfolding intermediates was discovered, which might reflect the possibility of subtle conformational variations at physiologically relevant temperatures. In this context, the low stability detected for this protein could be symptomatic of a high structural plasticity, which is necessary to interact with its molecular partners along the ERK-MAPK signaling pathway. In the future, we plan to apply state-of-the-art TROSY NMR experiments aimed at confirming the dynamics of the protein in different ranges of time scales at conditions where signal broadening and/or precipitation might. Our results complement the wealth of structural studies already present in the literature, with possible implications for the drug design of compounds to regulate MEK1, as well as for a more accurate description of the basic biological mechanisms in which it participates.

## Data Availability

The data are available from the authors upon reasonable request.

## Supplementary Materials

The following supporting information can be downloaded at: https://www.mdpi.com/article/10.3390/biom16060845/s1. Figure S1: Intrinsic fluorescence studies of MEK1 at different pH values. (A) The emission fluorescence spectra of MEK1 at different pH values at 25 °C after excitation at 280 nm. (B) The emission spectrum at 295 nm at pH 8.0. (C) Thermal denaturations followed by the emission at 330 nm, after excitation at 280 nm (the y-axis is scaled to allow for comparison among the different thermograms); Figure S2: Far-UV CD studies of MEK1 at different pH values. (A) The far-UV CD spectra of MEK1 at two pH values at 25 °C. (B) Thermal denaturations followed by the raw ellipticity at 222 nm (the y-axis is scaled to allow for comparison among the different thermograms); Figure S3: Chemical denaturations of MEK1 followed by fluorescence. The variation in the <λ> in the urea-denaturations (A) and GdmCl-denaturations (B) at 25 °C; Figure S4: Chemical denaturations of MEK1 followed by far-UV CD. The variation in the raw ellipticity at 5 μM (blue, blank squares) and 10 μM (red, blank circles) of MEK1 (in protomer units) at 5 °C.

## Abbreviations

The following abbreviations are used in this manuscript:

ANS	8-anilinonapthalene-1-sulfonic acid
ATP	adenosine triphosphate
CD	circular dichroism
CNA	constraint network analysis
DLS	dynamic light scattering
DSC	differential scanning calorimetry
ERK	extracellular signal-regulated kinase
GdmCl	guanidinium hydrochloride
ITC	isothermal titration calorimetry
MAPK	mitogen-activated protein kinase
PDB	protein data bank
PTM	post-translational modification
UV	ultraviolet
