# Supplementary material for: Unfolding Behavior and Conformational Changes Under Different Denaturing Conditions of MAPK 1 (MEK1)
Source: Biomolecules. 2026 Jun 9;16(6):845. doi: 10.3390/biom16060845 (PMC13296959; doi:10.3390/biom16060845)

Supplementary Material

Unfolding behavior and conformational changes under different denaturing conditions of MAPK kinase 1 (MEK1)

María Gabriela Álvarez-Rodríguez, Sonia Vega, Felipe Hornos, Adrian Velazquez-Campoy, Bruno Rizzuti and José L. Neira

Figure S1: **Intrinsic fluorescence studies of MEK1 at different pH values**. (A) The emission fluorescence spectra of MEK1 at different pH values at 25 ºC after excitation at 280 nm. (B) The emission spectrum at 295 nm at pH 8.0. (C) Thermal denaturations followed by the emission at 330 nm, after excitation at 280 nm (the y-axis is scaled to allow for comparison among the different thermograms).

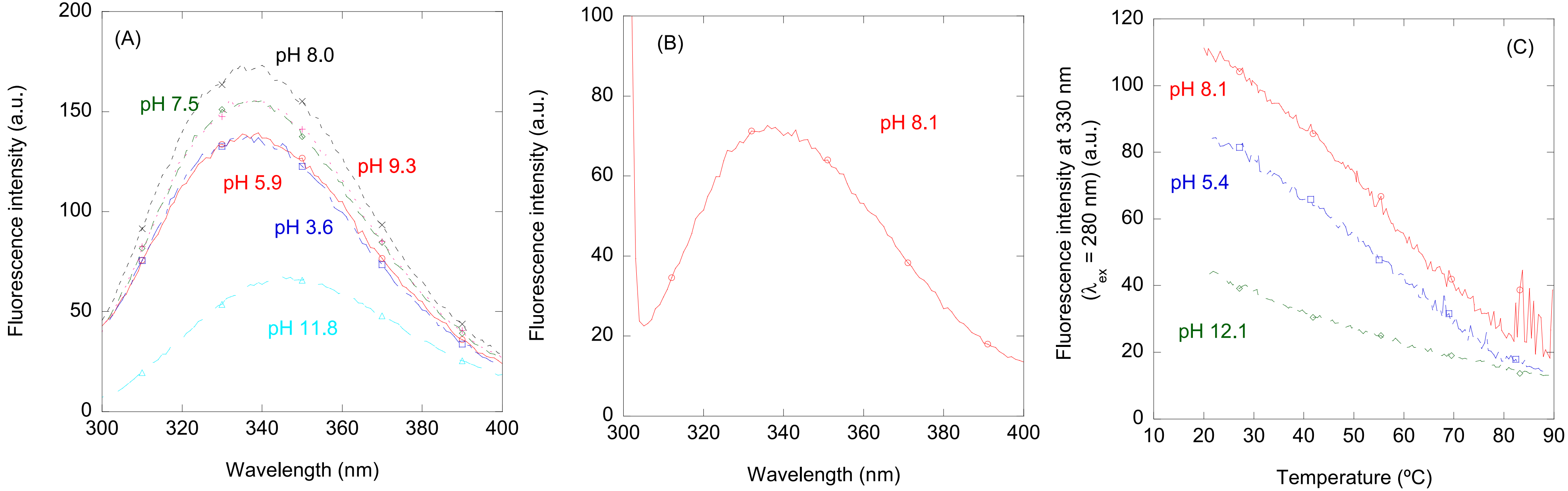

Figure S2: **Far-UV CD studies of MEK1 at different pH values**. (A) The far-UV CD spectra of MEK1 at two pH values at 25 ºC. (B) Thermal denaturations followed by the raw ellipticity at 222 nm (the y-axis is scaled to allow for comparison among the different thermograms).

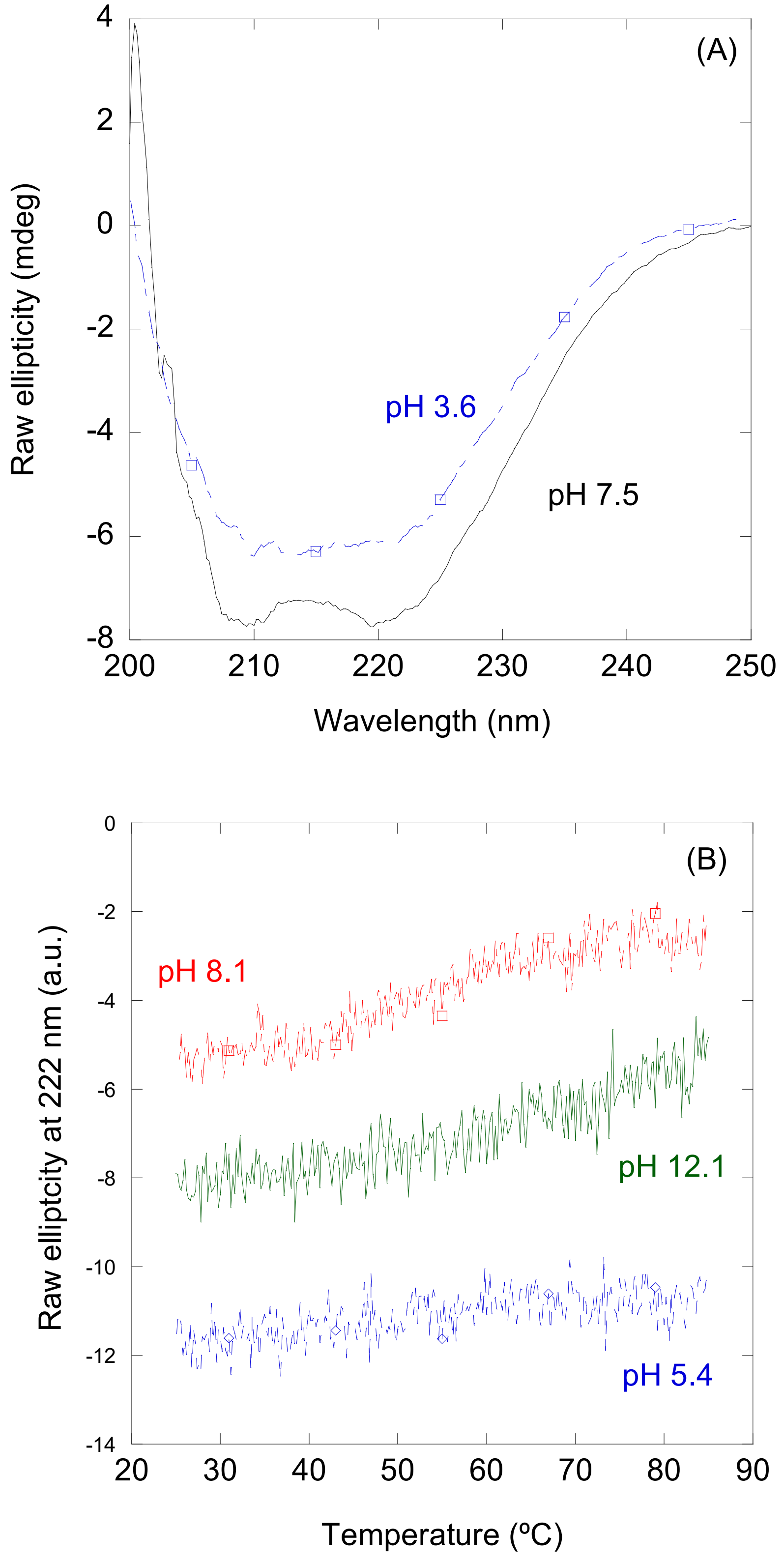

Figure S3: **Chemical denaturations of MEK1 followed by fluorescence.** The variation of the <λ> in the urea-denaturations (A) and GdmCl-denaturations (B) at 25 ºC.

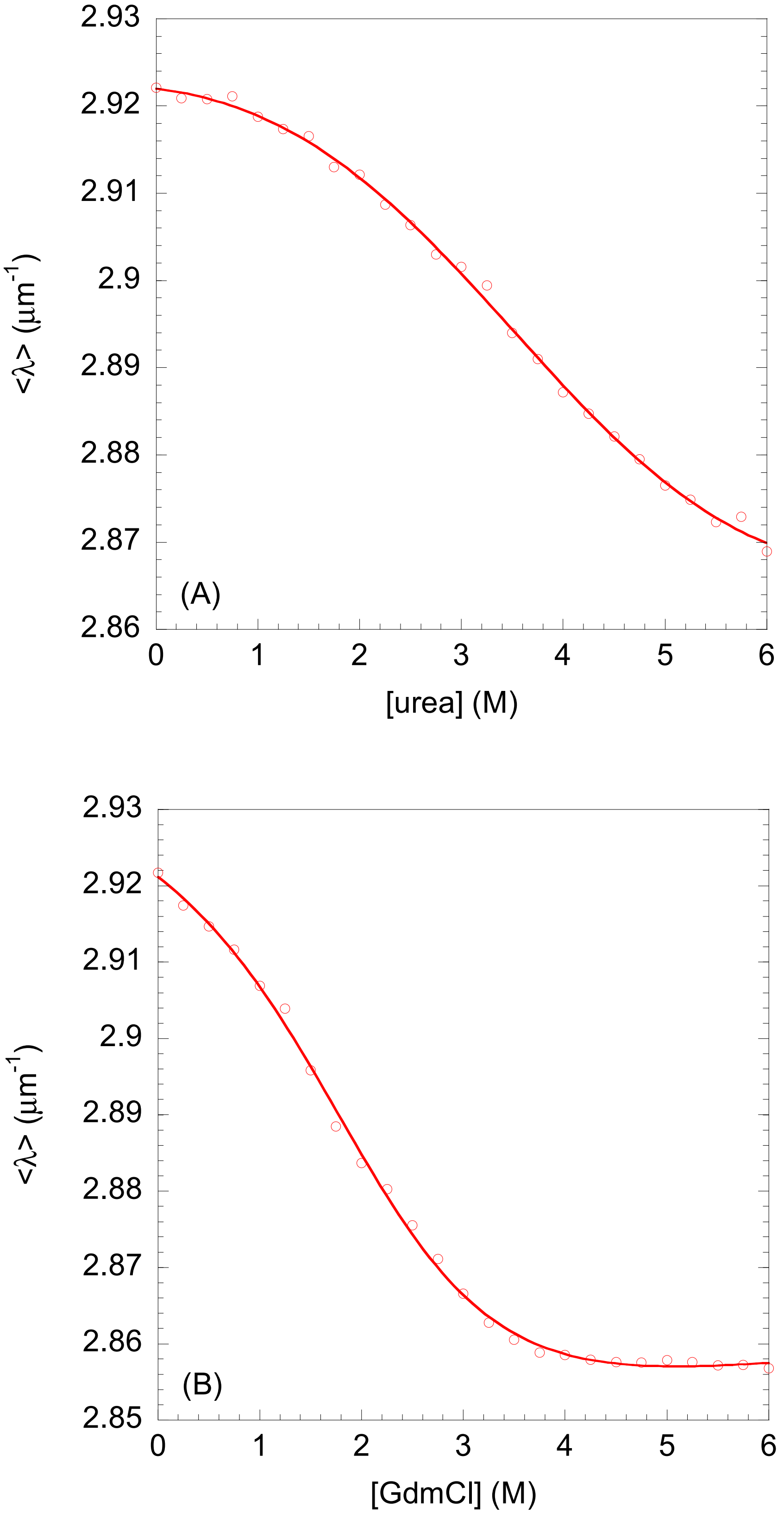

Figure S4: **Chemical denaturations of MEK1 followed by far-UV CD.** The variation of the raw ellipticity at 5 µM (blue, blank squares) and 10 µM (red, blank circles) of MEK1 (in protomer units) at 5 º C.

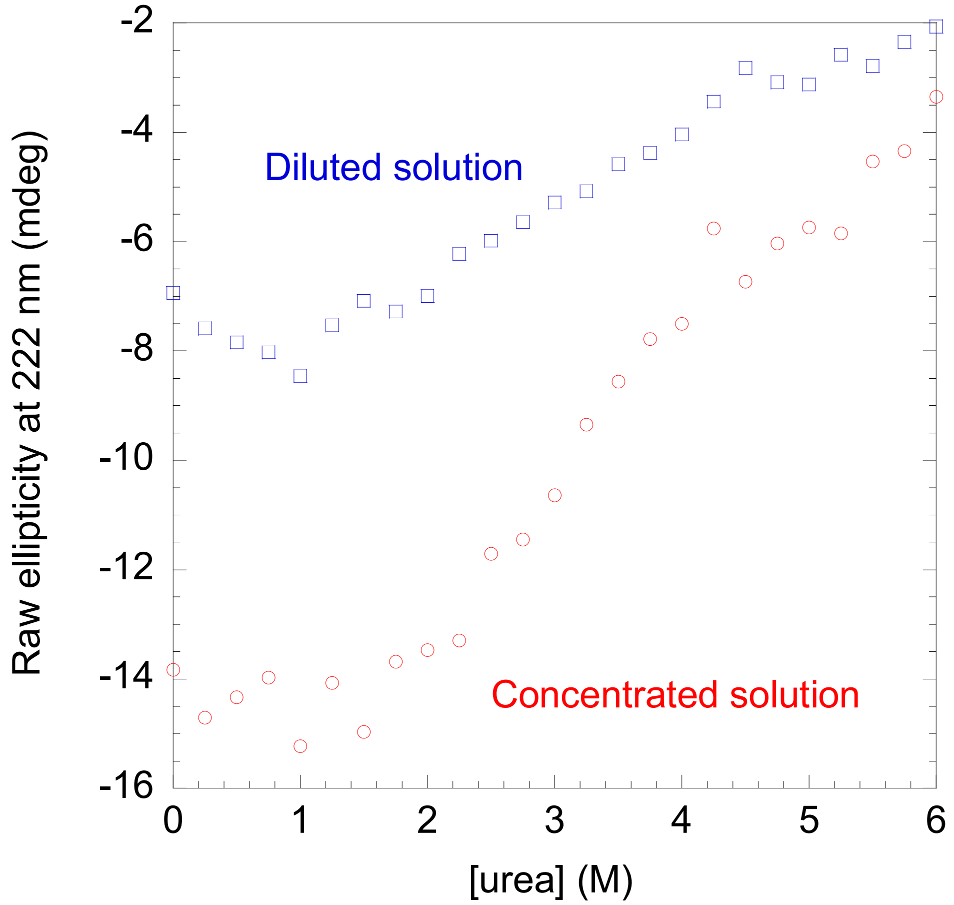

Supplement: Supplementary file 1 [file biomolecules-16-00845-s001.zip › biomolecules-4298446-supplementary.pdf]
